# TRONTIER 1 and TRONTIER 2: Pivotal trials of trontinemab in early symptomatic Alzheimer’s disease

**DOI:** 10.1002/alz70859_104294

**Published:** 2025-12-26

**Authors:** Janice Smith, Catherine J. Mummery, Jeffrey L. Cummings, Gil D. Rabinovici, Stephen Salloway, Reisa A. Sperling, Henrik Zetterberg, Angeliki Thanasopoulou, Christopher Lane, Paul Delmar, Gregory Klein, Ruth Croney, Jakub Wojtowicz, Carsten Hofmann, Luka Kulic, Hideki Garren

**Affiliations:** ^1^ Roche Products Ltd, Welwyn Garden City United Kingdom; ^2^ University College London, London, Greater London United Kingdom; ^3^ Chambers‐Grundy Center for Transformative Neuroscience, Kirk Kerkorian School of Medicine, University of Nevada, Las Vegas, NV USA; ^4^ Weill Institute for Neurosciences, University of California, San Francisco, San Francisco, CA USA; ^5^ Butler Hospital and Warren Alpert Medical School, Brown University, Providence, RI USA; ^6^ Massachusetts General Hospital, Harvard Medical School, Boston, MA USA; ^7^ Institute of Neuroscience and Physiology, The Sahlgrenska Academy at the University of Gothenburg, Mölndal Sweden; ^8^ F. Hoffmann‐La Roche Ltd, Basel Switzerland; ^9^ F. Hoffmann‐La Roche Ltd, Basel, Basel Switzerland; ^10^ Genentech, Inc., South San Francisco, CA USA

## Abstract

**Background:**

Developing potent therapies that reduce amyloid plaques has been shown as one means of producing clinical benefit in the early stages of symptomatic Alzheimer’s disease (AD). Trontinemab is a novel amyloid‐targeting Brainshuttle™ antibody specifically engineered for efficient transferrin receptor 1‐mediated transport across the blood–brain barrier. Trontinemab has demonstrated robust and rapid mean amyloid plaque removal of 107 centiloids after 28 weeks of 3.6 mg/kg treatment in the Phase Ib/IIa Brainshuttle AD study (n=12; NCT04639050), coupled with a low incidence of amyloid‐related imaging abnormalities (ARIA). Based on the results from the Brainshuttle AD study, to be presented at AAIC 2025 by Kulic et al., a pivotal program with trontinemab in early AD will be discussed.

**Method:**

TRONTIER 1 and 2 are two identically designed global, randomized, double‐blind, placebo‐controlled, parallel‐group Phase III studies designed to investigate the efficacy, safety, tolerability, pharmacokinetics (PK), and pharmacodynamics of trontinemab following intravenous infusion in participants with early AD who have confirmed amyloid pathology. A pre‐screener study, Traveller, based on a brief clinical assessment and a plasma biomarker will also be initiated to enable broader community outreach and extend access to these trials to more diverse populations.

**Result:**

The primary endpoint is the change from baseline on the Clinical Dementia Rating – Sum of Boxes at 18 months’ treatment duration. Secondary outcome measures include assessments of cognition, function, behavioral symptoms, and quality of life. PD effects of trontinemab will be evaluated using amyloid and tau positron emission tomography, magnetic resonance imaging, and fluid biomarkers. Additional important objectives include assessment of safety, PK, and immunogenicity.

**Conclusion:**

The recent Phase Ib/IIa interim results suggest that rapid and robust amyloid plaque clearance and fluid biomarker changes may be achieved with low ARIA incidence. The overall favorable safety and interim biomarker results to date support the rationale for moving into the pivotal Phase III studies TRONTIER 1 and TRONTIER 2. These studies will provide the opportunity to assess whether treatment with trontinemab slows disease progression in people living with early symptomatic AD.

